# Treating with minerals in the Middle Ages: the rare substance *mūmiyāʾ* (pitch-asphalt) and its medicinal uses in Byzantium

**DOI:** 10.1017/mdh.2024.25

**Published:** 2024-07

**Authors:** Petros Bouras-Vallianatos, Fabian Käs

**Affiliations:** 1 Department of History and Philosophy of Science, National and Kapodistrian University of Athens, University Campus, 15771 Athens, Greece; 2 Martin-Buber-Institute for Jewish Studies, University of Cologne, Albertus-Magnus-Platz, 50923 Köln, Germany

**Keywords:** Minerals, Pitch-asphalt, *Mūmiyāʾ*, Fractures, Byzantine medicine, Islamicate medicine

## Abstract

Premodern medicine used a variety of mineral substances for therapeutic purposes. The present article deals with pitch-asphalt, and, in particular, a precious kind of it called *mūmiyāʾ* originating in Persia. It was first described in detail in the Arabic pharmacological tradition, and its fame spread throughout the medieval Mediterranean, including Byzantium. By editing and examining for the first time a previously unexplored medieval Greek text on *mūmiyāʾ*, this study offers new insights into the medicinal uses of this substance. It also significantly increases our understanding of the intense cross-cultural transfer of medical knowledge from the Islamicate world to Byzantium by showing that this was not merely based on the translation of a few Arabic medical works into Greek, but was a multifaceted phenomenon involving a complex nexus of sources that require further investigation.

## Introduction

Various kinds of mineral substances played an important role in premodern therapeutics.^1^ Some of these, such as the famous Lemnian earth or Armenian bole, were often mentioned as being extremely effective for infectious diseases, and modern research has recently suggested that they potentially had a strong antibacterial effect.^2^ This article aims to cast light on a previously unknown episode in the long history of pitch-asphalt as a pharmacological ingredient by focusing on the reception of Islamicate pharmacological lore on this substance in Byzantium.^3^ It is divided into three parts. The first introduces pitch-asphalt as a mineral drug as evidenced in the works of ancient and medieval authors, with a particular emphasis on the variety of pitch-asphalt called *mūmiyāʾ* originating in Persia. The next part addresses the introduction and dissemination of medical knowledge about *mūmiyāʾ* in Byzantium, including evidence from several unpublished sources. The last section provides the first-ever edition and English translation of a medieval Greek opuscule on *mūmiyāʾ*, known as μουμιέ (*moumie*) or μώμιον (*mōmion*) in Greek.

## Pitch-asphalt in premodern medicine

Different kinds of the mineral substance asphalt (Greek: ἄσφαλτος, Latin: *bitumen*) were mentioned by Pliny (AD 23/4–79) and Dioscorides (fl. first century AD), including those originating in Judaea, Sidon, Babylon, the Ionian island of Zakynthos, and Apollonia, a place near the ancient city of Epidamnus, now Durrës in Albania.^4^ Dioscorides informs us that the best variety is found in Judaea and Pliny specifies that it is manufactured on the right bank of the Dead Sea.^5^ Dioscorides also refers to two more kinds: one from Phoenicia and a liquid form from Sicily. He calls the one from Apollonia pitch-asphalt (πισσάσφαλτος) because this particular one smells like a mixture of pitch^6^ and asphalt. According to Dioscorides, asphalt has anti-inflammatory, agglutinative, dispersive, and emollient properties, and is beneficial for uterine suffocations. It could also be mixed with other substances and used, either in the form of a potion or a clyster, for a large variety of ailments, including chronic coughs, asthma, breathlessness, snake bites, pains in the hip joint and the side, bowel ailments, and dysentery. Interestingly, it was also used to glue the hair and when mixed with wax and soda was used in the form of plaster for those suffering from gout. Pliny adds a couple more uses, including skin affections, such as *lepra* and lichen-like eruptions, and quartan fever; asphalt can also heal sinews.

These uses were more or less followed by other ancient authors, including Galen (AD 129–216/17). The Pergamene physician pays particular attention to the use of asphalt in closing up bleeding wounds, both as a simple and as the active ingredient in a special group of plasters, the so-called ‘barbarian haemostatic [plasters] made of asphalt’ (δι᾽ ἀσφάλτου ἔναιμοι βάρβαροι).^7^ His references were adopted by late antique physicians, including, for example, Aetios of Amida (fl. first half of the sixth century AD) and Paul of Aegina (late sixth century–d. after AD 642). In addition to the use of asphalt as an agglutinative, the latter authors particularly emphasised its efficacy in cases of dysentery and hydrophobia.^8^

Classical knowledge about asphalt was transferred to the Islamicate world through translations of the works of Galen and Dioscorides, in which the translators seem to have been puzzled as to how to render the Greek πισσάσφαλτος, often transcribed as *fīṭṭāsfalāṭūs* or *biṭṭasfalṭus.* ^9^ Identification of Dioscorides’ ‘pitch-asphalt’ with *mūmiyāʾ* is attested for the first time in the ninth-century standard translation of Dioscorides’ work by Iṣṭifān ibn Basīl.^10^ An older translation discovered only a few years ago (*vetus translatio*) simply transcribed the Greek term and translated it literally as *zift al-qufr* (‘pitch of asphalt’)^11^; the term *mūmiyāʾī* was applied in this text to Dioscorides’ ζώπισσα, thus adding another component to the import of ancient Greek knowledge on pitch-asphalt in the Islamicate medical tradition and showing a sort of uncertainty in correlating *mūmiyāʾ* with a particular Greek term.^12^ The revised version of Iṣṭifān’s translation by Ibn Sīnā’s teacher al-Nātilī (*fl.* eleventh century) identified again πισσάσφαλτος with *mūmiyāʾ*; the author added the literal translation *zift al-qufr.* The translators of the twelfth century did not actually equate πισσάσφαλτος and *mūmiyāʾ.* For example, al-Malaṭī gives the literal equivalent of the Greek term, i.e. *zift al-qufr*, and Mihrān only explains the Greek name as a ‘variety of asphalt (*qufr*) from Apollonia’.^13^ However, al-Rāzī (d. c. 925) cited Dioscorides’ very short description in his *Comprehensive Book on Medicine*,^14^ where he omitted any medicinal uses. This shows that he indeed identified *mūmiyāʾ* with πισσάσφαλτος. After him, many authors writing in Arabic on pharmacognosy, such as Ibn Samajūn, Ibn Wāfid, al-Idrīsī, al-Ghāfiqī, and Ibn al-Bayṭār, followed his model and cited Dioscorides’ passage in their entries on pitch-asphalt.^15^ In what follows, we will show that despite the efforts to identify *mūmiyāʾ* with varieties of asphalt or bitumen mentioned in Dioscorides and Galen, the way the use of the term was further developed in both the Islamicate and Byzantine traditions widely differed from the scarce ancient accounts.

The etymology of the Arabic form *mūmiyāʾ*—other common spellings include *mūmiyā* (without the perhaps hypercorrect *hamza*) and *mūmiyāʾī* (coming close to the alleged original Persian form)—is not entirely clear. It certainly depends on *mūm*, the Persian word for ‘wax’. The most probable origin would be the Persian *mūm-āʾīn* (‘like wax’).^16^ According to some less likely interpretations reported by the Iranian polymath Abū l-Rayḥān al-Bīrūnī (d. 1048), it may be composed of *mūm* and the Persian *āb* (‘water’) or even the toponym *Ābīn*, a place near Dārābjird (now Darab in Iran’s Fars Province)^17^. According to reports by Arab geographers and pharmacologists, *mūmiyāʾ* was found in Persia and other places such as Syria, Yemen, near Cordova in Spain, and on the Atlantic coast of Northern Africa. The Persian variety was considered of highest quality. Quoting from an anonymous book on geography entitled *Ashkāl al-aqālīm* (*The Shapes of the Climatic Zones*), al-Bīrūnī informs us that it originated in a rock cave in Persia. The cave was unsealed only once a year when a small amount was collected under the supervision of high-ranking officials and then delivered to the Sultan of Dārābjird:^18^

The author of the *Ashkāl al-Aqālim*…writes: *Momyāʾī* is found in Dārā Bijard in a cave. It is reserved for the king and the mouth of the cave is guarded by sentries. At a specified time each year officials, despatchers of letters and the courtiers of the king gather together and unseal the mouth of the cave. Pissasphalt collects within the crevice of a stone in the lower portion in the size of a pomegranate. It is sealed in the presence of these dignitaries and all the officials of the government take a little of it. This is the real pissasphalt. All the other pissasphalt varieties are counterfeit. There is a village near the cave known as *Ābīn.* The name *momyāʾī*, is, therefore, an eponym, and is mom *Ābīn* (i.e. the *Ābīn* wax).^19^

In what follows, al-Bīrūnī gives a few variants of this story and informs us about other places of origin in Iran and neighbouring countries. The text also praises the effectiveness of this mineral when applied externally in gluing bones back together and repairing fractures.

The anecdote concerning the cave predates al-Bīrūnī and his source. Already in the ninth century, the geographer Ibn al-Faqīh had said the same about a cave near the now ruined city of Arrajān on the border between what are today the provinces of Khuzestan and Fars in Iran. In his *Kitāb al-Buldān* (*Book on Countries*), he wrote that in this cave there was a well, in which the water changed over time into white *mūmiyāʾī.* The iron door of the cave was opened just once a year in the presence of local dignitaries, who then sent the *mūmiyāʾ* to the Sultan. Ibn al-Faqīh also says that an amount equal to one lentil administered orally with water heals fractures and similar conditions.^20^ The famous geographer Yāqūt al-Ḥamawī (1179–1229) repeats Ibn al-Faqīh’s information that the cave was in Arrajān; he also emphasises the use of *mūmiyāʾ* for bone fractures.^21^ It is an interesting fact that al-Bīrūnī and Ibn al-Faqīh relate almost the same story about two different towns, which makes one wonder whether this drug was actually that unique and if there was really such a monopoly. Centuries later, other authors locate the cave near Iṣṭakhr or Shīrāz in Fārs, and the German traveller Engelbert Kaempfer tells us the same story as late as in 1712.^22^ The tenth-century Syriac lexicographer Bar Bahlūl even stated that the real discoverer of the source of *mūmiyāʾ* was Daniel, the biblical prophet.^23^ Several authors report mineral *mūmiyāʾ* found in the Islamicate West, either in the environs of Cordova, Lorca, or Meknes. According to others, a stone is found in Yemen containing such a liquid. Especially interesting is the report of Muḥammad ibn Aḥmad al-Tamīmī (d. c. 980). He tells us that the Fatimid caliph al-Muʿizz had sent a missionary of Ismāʿīlī Shiism to what is now Algeria. A black mound he found on the seashore turned out to be a kind of *mūmiyāʾ*, with which he successfully treated the broken bones of a building worker.^24^

Arab authors of the early Middle Ages recommended mineral *mūmiyāʾ* for several purposes. Because of the vast number of entries dedicated to this substance in the Arabic medical literature, we focus here on the most important classical pharmacognostic texts, namely ʿAlī ibn Rabban al-Ṭabarī’s *Firdaws al-ḥikma* (*Paradise of Wisdom*, ninth century), al-Rāzī’s *al-Ḥāwī* (*Comprehensive Book on Medicine*, tenth century) and his *al-Kitāb al-Manṣūrī* (*Book for al-Manṣūr*),^25^ al-Majūsī’s *al-Kitāb al-Malakī* (*Royal Book*, tenth century), Ibn Sīnā’s *Qānūn fī l-ṭibb* (*Canon of Medicine*, eleventh century), and Ibn al-Bayṭār’s *al-Jāmiʿ li-mufradāt al-adwiya wa-l-aghdhiya* (*Collector of Simple Drugs and Foodstuffs*, thirteenth century).^26^ The main and most illustrious indication of natural *mūmiyāʾ* was the treatment of broken bones, and references to this can also regularly be found in the works by geographers and mineralogists mentioned above. It is worth noting that this use was never singled out so prominently in the earlier Greek tradition as far as pitch-asphalt is concerned. The second most important benefit was for coughing up blood. Other indications listed in these sources include headaches, migraine, facial paralysis, epilepsy, trembling and tetanus, earache and suppuration from the ears, angina, cough, hiccough, suffocation, conditions of the heart and spleen, incontinence, ulcers in the bladder and urethra, dropsy, jaundice, and pains in the nerves and joints. It was also recommended for poisons and scorpion stings. The drug was mostly administered orally and dissolved in diverse liquids. Furthermore, for some indications, it was dripped into the ears or the nostrils.

Over the next few centuries, there was a remarkable semantic expansion of the term *mūmiyāʾ* and its Latin counterpart *mumia*, which were also associated with the bituminous substance from embalmed mummies. For example, one of the most widespread works on simple drugs in Latin, the twelfth-century Salernitan treatise *Circa instans*, mentions *mumia* as a hot and dry substance, which is found in the tombs of people embalmed with spices. According to the text, the *mumia* is found near the brain and spine, is black, and has a bad smell; it is used as a haemostatic and also in composite drugs for dysentery, ailments of the intestines, and excessive bleeding in menstruation.^27^ *Mumia* is also found in the vast Byzantine pharmacopoeia *Dynameron* by the so-called Nicholas Myrepsos, which was composed by the late thirteenth century, and it is particularly defined as ‘blood of a dead person, called *moumia* by the Italians’.^28^

Arabic accounts of this ‘*mūmiyāʾ* from the graves’ (*mūmiyāʾ qubūriyya*) are actually rare.^29^ Ibn al-Bayṭār (1197–1248), in his highly influential *Jāmiʿ*, reports four kinds of *mūmiyāʾ*: the pitch-asphalt of Dioscorides, the asphalt of Judaea (*qufr al-Yahūd*), the above-mentioned stone from Sanaa (Yemen), and the substance obtained from embalmed corpses.^30^ According to Ibn al-Bayṭār, the latter was used by the Rūm (‘Romans, Greeks, or even Byzantines’) in ancient times. The Jewish philosopher Mūsā ibn Maymūn (a.k.a. Maimonides, d. 1204), who spent a significant part of his life in Egypt, mentioned in his entry on *mūmiyāʾī* only the variety from the graves.^31^ The oldest medical source describing this type of ‘mummy found in graves’ is actually Ibn al-Jazzār (d. c. 980) from Kairouan in his book on simple drugs.^32^ He apparently depends on his teacher Isḥāq ibn ʿImrān. Lastly, al-Tamīmī’s account is also highly interesting. He stated that the Pharaohs used to fill the corpses with the mineral ‘mummy from the West’ (*mūmiyāʾ Maghribī*), as he calls it.^33^

The use of *mūmiyāʾ* from Egyptian mummies became extremely widespread all over the Mediterranean and beyond in the Middle Ages. There were apothecary jars full of it until at least the sixteenth and in some cases even into the nineteenth century. Various stories survive about the actual origin of this ingredient, especially after the fifteenth century, when there was a shortage of ancient Egyptian mummies and merchants quite often acquired the bodies of recently dead persons that had been prepared for this purpose by local experts.^34^

## *Mūmiyāʾ* in Byzantine texts

Having briefly sketched out the story of the mineral *mūmiyāʾ* and the closely related bituminous substance from mummies, we can now focus on the transmission of the knowledge of this ingredient in Byzantium. A letter that has survived from the ninth century provides us with a unique testimony to relations between the Byzantine and Islamicate courts and also concerning the role played by rare drugs as diplomatic gifts.^35^ It also attests to the gradual introduction of Islamicate pharmacological knowledge to Byzantium, although not in the form of a translation of a single work from Arabic into Greek, as in the case of, for example, Ibn al-Jazzār’s (*fl.* tenth century) *Zād al-Musāfir wa-qūt al-ḥāḍir* (*Provisions for the Traveller and Nourishment for the Sedentary*) and al-Rāzī’s (d. c. 925) *Kitāb fī l-Judarī wa-l-ḥaṣba* (*Treatise on Smallpox and Measles*), which became available in Greek by the early twelfth century.^36^ This letter is from the son of the Fatimid Caliph to Romanos (later Romanos II, sole r. 959–63), son of Byzantine Emperor Constantine VII (sole r. 945–59), and is connected with the frequent embassies between the two states in the late 950s.^37^ The epistle was accompanied by a gift in the form of a precious substance, an invaluable sort of panacea drug, the rare mineral called μουμιέ (*moumie*) in Greek.

The text gives a lot of information about this *mūmiyāʾ.* It was given in a small silver vessel, is characterised as ‘priceless’ (ὑπέρτιμον) and ‘more precious than precious gems’ (τῶν τιμίων λίθων τιμιώτερον), and is indicated as most efficacious for the ‘treatment of the broken limbs of humans and birds’ (θεραπείαν συντριβῆς μελῶν ἀνθρώπων τε και πτηνῶν),^38^ which is the most important use of the substance in both the Arabic and the Byzantine traditions. The letter informs us that this substance was only available to the Fatimid Caliph in Cairo and the Caliph of Baghdad, thus implying that it was not sold on the open market. It does not provide accurate details about the place of origin, apart from the fact that the substance originated in a guarded ‘rock which oozes droplets like tears’ (ἐπὶ πέτρᾳ καὶ στάζει ὡς δάκρυον), which is in line with the story about the rock cave in Persia that appears in the Arabic sources as early as the ninth century. Interestingly, the text states that the mineral is not found in any other places in East and West, and it reports that most recently they had only been able to collect two litres^39^ (~640g) of *mūmiyāʾ* from the guarded ‘rock’ and that they had also found some quantity of this substance on the seashore; the latter, when drunk, was even more efficacious for broken legs, arms, and ribs.^40^ This kind of information is not found in any currently available Arabic source.

Next come details about its use and dosage. According to the text, a quantity of one silver *miliarision* ^41^ should be dissolved in a little oil and swallowed.^42^ The text also includes a procedure for testing it. To be specific, it is recommended that a bird or an animal that has a broken limb should drink some of it, and the fracture should be bandaged for three or four days; after this period, the animal should walk normally once the bandage is untied.^43^ As we will see below, similar forms of testing appear in Arabic sources as well. *Mūmiyāʾ* is also recommended for the treatment of catarrh, if the body becomes cold and full of humours (κατάρροιαν τὴν ἀπὸ ψύξεως γενομένην, καὶ εἰς τοὺς χυμούς), epilepsy (ἐπιληψίας), loss of sight (σκοτασμούς), speech impediment (μογιλάλον), cough (βῆχαν), sore throat (κυνάγχην), spleen affections (σπλῆνα), and scorpion bite (δηχθῇ τις ὑπὸ σκορπίου).^44^ The references to epilepsy, spleen affections, and the scorpion bite clearly belong to the expansion of the use of pitch-asphalt in the Arabic tradition, as we saw above. The letter ends with a further emphasis on how unique and invaluable *mūmiyāʾ* is and mentions an adulterated version of *mūmiyāʾ*, viz. a mixture of pitch with other substances, which was often sold by merchants but, when tested, has been shown not to be as efficacious as true *mūmiyāʾ.* ^45^

The absence of translations or adaptations of Arabic works on simple drugs in medieval Greek, apart from a work on purgative drugs ascribed to St. John of Damascus,^46^ meant that *mūmiyāʾ* was not a particularly popular ingredient in Byzantine medical literature, and there are only a few mentions of it. The first reference is found in the *Ephodia tou Apodēmountos*, the Greek translation of the aforementioned Arabic treatise, *Zād al-Musāfir wa-qūt al-ḥāḍir* by Ibn al-Jazzār.^47^ It appears as an ingredient in a composite drug for the treatment of bleeding from the ears. The earliest surviving witness of the text that retains this particular chapter, the twelfth-century Parisinus gr. 2311 (f. 59r, l. 28), reads μούμιεν,^48^ which is very close to the term in the letter that we saw above and also to the original Arabic word. In fact, the Greek term has the diphthong *ou* for the Arabic letter *wāw*, while the combination of *yāʾ* and *alif* at the end have been rendered as *ie* with the use of the Greek letters *iota* and *epsilon.* The final *nu* in Greek here might point to the abovementioned Arabic variant *mūm-āʾīn.*

The next two references in Byzantine Greek retain the term *mōmion*, which is the same term as is used for the mineral substance in the anonymous Byzantine work that is edited below. Moreover, here we can see a distinct process of Greekification in the use of the ending -*on*, which makes the term neuter in gender, and the rendering of *wāw* with *omega* in the first syllable, making the entire word more euphonic in Greek. The first mention is found in an anonymous veterinary work on birds, which is dedicated to an emperor called Michael, most probably Michael VIII (r. 1282–1328), in which consumption of *mōmion* along with meat is recommended for fractures.^49^ The second is in the unpublished recipe book of Benjamin the Jew, where *mōmion* is used as an ingredient in a composite drug, a plaster for testicular rupture.^50^ The third reference comes from another unpublished recipe book by the otherwise unknown medical author called Andreiomenos. The text retains the term *moumia*; the substance is recommended as a simple drug, which should be swallowed in the form of a powder for the treatment of dysentery.^51^ It is not clear whether it refers to the mineral *mūmiyāʾ* or the bituminous substance from mummies. It is worth noting that Andreiomenos’s recipe book contains some recipes derived from Latin *antidotaria*, in which the term *mumia* is mainly used with reference to human mummies. The term *moumia* also appears, as we saw above, in the *Dynameron*, with reference to the blood of a dead person.

The most detailed treatment of *mūmiyāʾ* is found in an anonymous opuscule, which is edited and translated as part of this article. The text survives in two manuscripts, Vaticanus gr. 282 (=**V**), f. 444v, and Parisinus gr. 2194 (=**P**), f. 436r. It was first mentioned with reference to **P** by Charles du Cange in 1688, who also transcribed the first few sentences, without providing any other data about the term *mōmion.* ^52^ Since then, it has not been examined. Here *mōmion* clearly refers to the Persian variety of pitch-asphalt (*mūmiyāʾ*), which originates in rock caves (ll. 3–4) in line with the accounts in Arabic medical literature and the Fatimid letter discussed above. According to this text, the mineral is also found in other places, including Byzantium, but that sort is not of good quality (ll. 4–6); this probably refers to other kinds of asphalt, as we mentioned above in the cases of Pliny and Dioscorides.^53^ It is also clearly specified in the text that ancient Greek authors were not aware of *mūmiyāʾ* (ll. 2–3). The best variety is the one that is neither soft nor too hard and is black in colour (ll. 6–7). There is a strong emphasis in the text on the use of the mineral substance for fractures, which was also emphasised in the Islamicate pharmacological tradition and the Fatimid letter.

A process is suggested for testing the efficacy of *mūmiyāʾ* when treating birds with a broken leg, which is very similar to the corresponding content in the Fatimid letter and alludes to reports in the oriental mineralogical and geographical literature. In this case, the text refers to a small chicken, which, as noted in the Fatimid letter, should walk normally again in three or four days if the *mūmiyāʾ* is genuine (ll. 7–9). Such a test, involving a chicken, was also mentioned by al-Bīrūnī.^54^ In line with the Fatimid letter, it is recommended here that *mūmiyāʾ* should be taken as a sort of potion, not applied externally. In the Arabic tradition, we can see mentions of both internal and external application.^55^ Interestingly, the opuscule referring to the treatment of limb fractures gives details about different dosages according to the age of the patient (infants, 5-year-old children, adults, old men) and the seasons of the year (winter, summer), something that is missing in the Arabic pharmacological texts. *Mūmiyāʾ* should be dissolved in warm unmixed wine or in breast milk for infants, rather than in oil as in the Fatimid letter (ll. 10–13).

Lastly, in this text, it is also used for swollen genitals ὀγκωθεῖεν τὰ αἰδοῖα caused by a kind of flatulence/gas (πνεῦμα) in both children and adults (ll. 13–14), an indication that is not mentioned explicitly in any other texts dealing with *mūmiyāʾ.* ^56^ The only linguistic parallel (αἰδοῖα…ὀγκωθῆναι) in Greek is found in three miracles of St. Artemios that describe the miraculous treatment of patients.^57^ In fact, this condition is very rare in medical texts. It alludes to the so-called pneumatocele (πνευματοκήλη) of Paul of Aegina, a sort of aneurysm of the testicular artery.^58^ Paul warns that according to the first-century AD surgeon Leonides, an operation for pneumatocele could be fatal due to the risk of haemorrhage, but he nevertheless gives details about the procedure.^59^ Interestingly, the same condition is also briefly described by Leo the Physician (ninth[?] century) in his *Synopsis of Medicine* where he emphasises that it mostly occurs in children and should be treated with drying medications, not with surgery.^60^ He suggested two plasters, the so-called *Athēna* (Ἀθηνᾶ) and *barbara* (βαρβάρα), which, along with a large number of other ingredients, also include pitch,^61^ a substance similar in nature to *mūmiyāʾ.*

## Conclusion

By focusing on the reception of the mineral *mūmiyāʾ* in Byzantium, this study has had some important outcomes. At a strictly textual level, it has been shown how critical it is to edit and make widely available even brief texts dealing with Byzantine medical material. In this case, evidence from a previously unexplored medieval Greek opuscule on *mūmiyāʾ* along with details coming from other unedited Byzantine sources has allowed us to outline the reception and dissemination of knowledge concerning this medicinal substance in the Byzantine world. The latter is particularly important for studying the impact of the significant transfer of medical knowledge from the Islamicate world to Byzantium and shows that scholars should move beyond studying the translations of single works from Arabic into Greek, of which in any case there were very few, and explore the actual diffusion of Islamicate medical lore in Byzantine medical works.

Another notable conclusion is that the Byzantines were keen on supplementing their medical cabinet with material on diseases and substances that did not exist in Greek. *Mūmiyāʾ* was often connected with various kinds of Dioscoridean pitch-asphalt in the Islamicate world, and although Byzantines were familiar with the Mediterranean pitch-asphalt that featured in earlier Greek and Byzantine accounts, they were eager to show awareness of the unique variety of *mūmiyāʾ* originating in Persia; they also used a newly coined term in Greek, first *moumie* and later the Greekified version *mōmion.* Thus, when looking at the reception of Islamicate medical lore in Byzantium, we must realise that the main intention of the Byzantines was to provide complementarity with those texts already available in ancient and medieval Greek.^62^ The latter is also suggested by the fact that this particular kind of *mūmiyāʾ* was recommended especially for fractures of bones, a use that was never emphasised in the earlier Greek and Byzantine medical accounts of pitch-asphalt.

Moreover, there is the medical and pharmacological context. *Mūmiyāʾ*, although rare, was a therapeutic agent with a reportedly high efficacy, which had defined pharmaceutical applications for particular ailments, especially broken limbs. The Byzantine sources give unique information about the quantity of the substance that could be collected every year, expand the reported uses of the substance for other ailments (e.g. swollen genitals), and provide detailed data on dosage for particular age groups, thus making the Byzantine evidence a unique witness to the history of *mūmiyāʾ* in the Middle Ages. The constant emphasis on the process of testing is consistent with the pharmacological experimentation that is reported throughout the corpus of late Byzantine pharmacological works, thus connecting theory with practice.^63^

Lastly, we should mention the non-medical dimension, as evidenced in the letter to the son of the Byzantine emperor, which can provide an excellent way to explore how pharmacological knowledge had to be able to adapt to contexts outside medical practice. Drugs were often considered extremely prestigious from a cultural point of view and travelled long distances in the form of diplomatic gifts sent between rulers, thus giving another perspective on the study of the wider Mediterranean pharmacological tradition.

## Footnotes

1 On minerals in premodern medicine, see Dietlinde Goltz , Studien zur Geschichte der Mineralnamen in Pharmazie, Chemie und Medizin von den Anfängen bis Paracelsus (Wiesbaden: F. Steiner, 1972).

2

Effie Photos-Jones et al., ‘Greco-Roman Mineral (Litho)Therapeutics and their Relationship to their Microbiome: The Case of the Red Pigment *Miltos*
’, Journal of Archaeological Science: Reports, 22 (2018), 179–9230775415 10.1016/j.jasrep.2018.07.017PMC6360534; and Danae Venieri et al. ‘Bridging the Gaps: Bole and Terra Sigillata as Artefacts, as Simples and as Antibacterial Clays’, Minerals, 10.4 (2020), 348 32724664 10.3390/min10040348PMC7115821.

3 On the introduction and dissemination of Islamicate pharmacological lore to Byzantium, see Petros Bouras-Vallianatos , ‘Cross-Cultural Transfer of Medical Knowledge in the Medieval Mediterranean: The Introduction and Dissemination of Sugar-Based Potions from the Islamic World to Byzantium’, Speculum 96 (2021), 963–1008 35928988 10.1086/715838PMC7613226.

4 Pliny the Elder, *Natural history*, 35.51, in Karl Friedrich Theodor Mayhoff (ed.), *C. Plini Secundi Naturalis Historiae*, vol. 5 (Leipzig: Teubner, 1897), 297.3–299.2; Dioscorides, *De materia medica*, 1.73, in Max Wellmann (ed.), Pedanii Dioscuridis De Materia Medica, vol. 1 (Berlin: Weidmann, 1907), 72.12–73.22 . See also Celsus, *On Medicine*, 3.27.2, 5.3, 5.11, 5.19.2, 5.19.6, 5.19.20, 5.20.1, in Friedrich Marx (ed.), A. Cornelii Celsi (Leipzig and Berlin: Teubner, 1915), 143.13–14, 191.11, 193.7, 201.20, 202.1, 206.9.

5 Pliny the Elder, *Natural History*, 6.26, in Mayhoff (ed.), *op. cit.* (note 4), vol. 1, 1892, 472.8–9.

6 Pitch is the resin exudation collected from pine trees. See Dioscorides, *De materia medica*, 1.72, in Wellmann (ed.), *op. cit.* (note 4), vol. 1, 70.15–72.12.

7 Galen, *On the Capacities of Simple Drugs*, 6.30 and 11.2, in Karl Gottlob Kühn (ed.), Claudii Galeni Opera Omnia, vols 11 and 12 (Leipzig: Knobloch, 1826), 825.5 and 375.4–18. Galen, *On the Composition of Drugs According to Kind*, 2.22, *idem*, vol. 13, 1827, 555.13–561.13.

8 Aetios of Amida, *Tetrabiblos*, 2.49, in Alessandro Olivieri (ed.), Aetii Amidenii Libri medicinales, vol. 1 (Leipzig and Berlin: Teubner, 1935), 170.27–171.14 . Paul of Aegina, *Epitome*, 7.3.1, in Johan Ludvig Heiberg (ed.), Paulus Aegineta, vol. 2 (Leipzig and Berlin: Teubner, 1924), 198.18–19.

9 See the comprehensive analysis on the term with references to primary sources on the mineral substance by Fabian Käs , Die Mineralien in der arabischen Pharmakognosie: eine Konkordanz zur mineralischen Materia medica der klassischen arabischen Heilmittelkunde nebst überlieferungsgeschichtlichen Studien, vol. 2 (Wiesbaden: Harrassowitz, 2010), 1064–70. Cf. Albert Dietrich, ‘Mūmiyāʾ’, in P. Bearman et al. (eds), Encyclopaedia of Islam, Second Edition, vol. 7 (Leiden: Brill, 1993), 556 ; online version: https://referenceworks.brillonline.com/entries/encyclopaedia-of-islam-2/mumiya-SIM_5495?s.num=0&s.f.s2_parent=s.f.book.encyclopaedia-of-islam-2&s.q=mumiya (accessed 9 August 2023). Albert Dietrich , Dioscurides Triumphans. Ein anonymer arabischer Kommentar (Ende 12. Jahrh. n.Chr.) zur Materia medica, vol. 1 (Göttingen: Vandenhoeck & Ruprecht, 1988), 120 ; Albert Dietrich , Die Dioskurides-Erklärung des Ibn al-Baiṭār. Ein Beitrag zur arabischen Pflanzensynonymik des Mittelalters (Göttingen: Vandenhoeck & Ruprecht, 1991), 61 .

10 For the Arabic translations, see Käs, *op. cit.* (note 9), vol. 2, 1064 as well as Manfred Ullmann , Wörterbuch zu den griechisch-arabischen Übersetzungen des 9. Jahrhunderts; Supplement. Band II: Π‑Ω (Wiesbaden: Harrassowitz, 2007), 118f.

11 This anonymous text is the earliest Arabic translation and was identified by Manfred Ullmann, who analysed it in depth in his monograph: *Untersuchungen zur arabischen Überlieferung der* Materia medica *des Dioskurides* (Wiesbaden: Harrassowitz, 2009), 299 and 279. *Qufr*, or else *kufr, qufr al-Yahūd* and *kufr Yahūdī*, is the usual Arabic equivalent of the Greek ἄσφαλτος. The plural *aqfār* was sometimes used as a generic name for such varieties of fossil oil. The Arabic medical sources did actually strictly differentiate between *qufr* (Käs, *op. cit.* (note 9), vol. 2, 886–901), *mūmiyāʾ*, and *nafṭ* (νάφθα; Käs, *op. cit.* (note 9), vol. 2, 1087–94). The lexicographers of Classical Arabic mostly use the variant vocalisation *qafr* (e.g. Reinhard Dozy, *Supplément aux dictionnaires arabes*, vol. 2 (Leiden: Brill, 1881), 391). We would like to thank the anonymous reviewer for drawing our attention to this point. Since the term derives from the Aramaic *kufrā* and the Akkadian *kupru*, we preferred, however, the spelling *qufr/kufr* (see Manfred Ullmann et al., Wörterbuch der klassischen arabischen Sprache, vol. 1 (*kāf*), (Wiesbaden: Harrassowitz, 1970), 265a ).

12 Ζώπισσα is a much-debated term as far back as Dioscorides, *De materia medica*, 1.72, in Wellmann (ed.), *op. cit.* (note 4), vol. 1, 72.9–12; Lily Y. Beck (tr.), Pedanius Dioscorides of Anazarbus. De materia medica (Hildesheim: Olms–Weidmann), 57 : ‘Some say that *zōpissa* is the resin that is scraped with the wax from ships, which some call *apochyma*, since it is able to dissolve because it is bathed in sea water; others, however, call by this name the resin of the *pitys* pine’.

13 Dioscorides, *De materia medica, vetus translatio arabica*, MS Istanbul, Ayasofya 3704, f. 12r, l.10; Abū Sālim al-Malaṭī, Arabic translation of *De materia medica*, Parisinus arabe 4947, f. 12v, l. 4; Mihrān b. Manṣūr b. Mihrān, Arabic translation of *De materia medica*, MS Mashhad, Maktaba Riḍawiyya 5079 ṭibb 490, f. 46v.

14 Al-Rāzī, *Kitāb al-Ḥāwī fī l-ṭibb* (*Comprehensive Book on Medicine*), vol. 21 (Hyderabad: Dāʾirat al-Maʿārif al-ʿUthmāniyya, 1388/1968), 496ff. (s.v. *mūmiyāʾī*). See also Ibn al-Bayṭār, *al-Jāmiʿ li-mufradāt al-adwiya wa-l-aghdhiya* (*Collector of Simple Drugs and Foodstuffs*), vol. 4, (Būlāq, 1291 [1874]), 169f.

15 For these references, see Käs, *op. cit.* (note 9), vol. 2, 1065f.

16 This etymology is already to be found in Johann August Vullers’ *Lexicon persico-latinum etymologicum*, vol. 2 (Bonn: Impensis A. Marci, 1864), 1231a. The Syriac lexicographers preserved a form with the Middle Persian ending *-ag* (instead of *-ā*), which certainly predates the Arabic witnesses (Käs, *op. cit.* (note 9), vol. 2, 1068). Vullers also mentioned the Greek form ‘μουμία’ in this context. It has, however, become clear in the meanwhile that this Byzantine term is actually based on the Perso-Arabic forms.

17 Al-Bīrūnī, Abū l-Rayḥān, *Kitāb al-Jamāhir fī maʿrifat al-jawāhir (Most Comprehensive Book in Knowledge on Precious Stones)*, in Fritz Krenkow (ed.), (Hyderabad: Dāʾirat al-Maʿārif al-ʿUthmāniyya, 1355 [1936]), 204. Hakim Mohammed Said (tr.), *Al-Beruni’s Book on Mineralogy. The Book Most Comprehensive in Knowledge on Precious Stones* (Islamabad: Pakistan Hijra Council, 1410 [1989]), 176. Gotthard Strohmaier , Al-Bīrūnī. In den Gärten der Wissenschaft (Leipzig: Reclam, 1991), 224 .

18 This process recalls the ritualistic digging out of Lemnian earth on the Greek island of Lemnos, which used to take place once a year up to the early modern era. Galen, *On the Capacities of Simple Drugs*, 9.2, in Kühn (ed.), *op. cit.* (note 7), vol. 12, 168.9–178.14.

19 Al-Bīrūnī, *Jamāhir*, in Krenkow (ed.), *op. cit.* (note 17), 204.11–16; Said (tr.), *op. cit.* (note 17), 176. In al-Rāzī’s monograph on *mūmiyāʾ* (see below, note 25), the village where the drug was found was referred to as Ābān. The same text includes a variant of the story of the cave near that village. According to this version, it was discovered in the days of Persian mythical King Afrīdūn by a hunting soldier.

20 Ibn al-Faqīh, *Mukhtaṣar Kitāb al-Buldān (The Abridgment of the Book on Countries)*, in Michael Jan de Goeje (ed.), (Leiden: Brill, 1885), 199.12–200.3; see also Miguel Ángel González Manjarrés, ‘Presencia de mumia en la medicina medieval (siglos XI-XIV)’, in A. Paravicini Bagliani (ed.), Terapie e guarigioni (Florence: SISMEL, 2010), 163–97, esp. 167–8.

21 Yāqūt al-Ḥamawī, *Kitāb al-Buldān (Book of Countries)*, in Ferdinand Wüstenfeld (ed.), Muʿjam al-buldān, vol. 1 (Leipzig: Brockhaus, 1866), 194,16, s.v. ‘Arrajān’. He explicitly mentions Ibn al-Faqīh, whose wording he copied.

22 For the diverse medieval sources mentioned in the passage that follows, see Käs, *op. cit.* (note 9), vol. 2, 1069f. Kaempfer describes the tradition of collecting mineral mummy once a year in a cave near Dara in Persia. According to him, this tradition was resumed only in the seventeenth century after a gap of some centuries. On this, see note 54, below.

23 Rubens Duval (ed.), *Lexicon syriacum auctore Hassano bar Bahlule e pluribus codicibus edidit et notulis instruxit* (Paris: 1890, reprinted in Amsterdam: Philo Press 1970), vol. 1, col. 1033,13: *mwmʾyg … mūmiyā yurwā anna Dāniyāla l-nabiyya awwalu mani stakhrajahū bi-Fārisa wa-ʿallamahumu stikhrājahū.*

24 Al-Tamīmī, *al-Murshid fī jawāhir al-aghdhiya wa-quwā l-mufradāt min al-adwiya* (*The Guidebook to the Substances of Foodstuffs and the Properties of Simple Drugs*) (Maq. XI–XIV), Parisinus arab. 2870, f. 23r.

25 Al-Rāzī also wrote a short monograph on *mūmiyāʾ* entitled *Risāla fī Ṣifat al-mūmiyāʾ wa-manāfiʿihī* (*Epistle on the Description and Medicinal Uses of Pissasphalt*). See Fuat Sezgin, *Geschichte des arabischen Schrifttums*, vol. 3 (Leiden: Brill 1970), 290, no. 51. Ignacio Sánchez (University of Castilla-La Mancha, Escuela de Traductores de Toledo) is currently preparing a paper on this unique and hitherto neglected text. We are grateful to him for sharing his draft translation of the section on the origin of *mūmiyāʾ* with us.

26 Al-Ṭabarī, *Firdaws al-ḥikma (Paradise of Wisdom)*, in Muḥammad Zubayr al-Ṣiddīqī (ed.), (Berlin: Buch- und Kunstdruckerei ‘Sonne’, 1928), 405f.; al-Rāzī, *al-Ḥāwī*, *op. cit.* (note 14), vol. 21, 496; al-Rāzī, *al-Kitāb al-Manṣūrī (Book for al-Manṣūr)*, in Ḥasan al-Bakrī al-Ṣiddīqī (ed.), (Kuwait, 1408 [1987]), 177; al-Majūsī, *al-Kitāb al-Malakī* (*Royal Book*), vol. 2 (Būlāq, 1294 [1877]), 133; Ibn Sīnā, *Qānūn fī l-ṭibb* (*The Canon of Medicine*), vol. 1 (Būlāq, 1294 [1877]), 367; Ibn al-Bayṭār, *al-Jāmiʿ*, *op. cit.* (note 14), vol. 4, 169f. For more sources, see also Käs, *op. cit.* (note 9), vol. 2, 1064–67; and González Manjarrés, *op. cit.* (note 20), 167–75.

27
*Circa Instans*, *Liber de simplici medicina secundum Platearium* (Venice, 1497), 202va-b. See also the entry by Simon of Genoa, *Clavis sanationis (Key of Healing)*, in Barbara Zipser (ed.), *Simon Online*, s.v. mumia, at http://www.simonofgenoa.org/index.php?title=Mumia (accessed 9 August 2023). For references to a large number of Latin texts and relevant earlier bibliography on the topic, see González Manjarrés, *op. cit.* (note 20), 175–91. On manuscript illustrations of *mumia* in which an open tomb usually displays its contents, see Michael Camille , ‘The corpse in the garden: *Mumia* in medieval herbal illustrations’, in C. Chène (ed.), Il cadavere. The Corpse (Florence: SISMEL, 1999), 297–318 .

28 [Nicholas Myrepsos], *Dynameron*, 1.12a, in Ilias Valiakos (ed.), Nikolaos Myrepsos’ Dynameron. Critical Edition (Heidelberg: Propylaeum, 2020), 33.10–11: αἷμα ἀνθρώπου τεθνεῶτος, τὸ ἐπονομαζόμενον παρ᾽ Ἰταλοῖς, μούμια. The ingredient is one of many in the antidote *athanasia* for dysentery and excessive bleeding in menstruation. The same recipe is also found in the twelfth-century Latin *Antidotarium Nicolai*, 4, in Wouter S. van den Berg (ed.), Eene middelnederlandsche vertaling van het Antidotarium Nicolai: (Ms. 15624-15641, Kon. Bibl. te Brussel) met den latijnschen Tekst der eerste gedrukte Utg. van het Antidotarium Nicolai (Leiden: Brill, 1917), 11 , but without the explanation about this ingredient that features in the *Dynameron.* The same ingredient appears in one more composite drug in *Dynameron*, a kind of potion called *drosaton*, for dysentery and bowel ailments, 8.17, *idem*, 331.19–332.8.

29 Käs, *op. cit.* (note 9), vol. 2, 1068f.

30 Ibn al-Bayṭār, *Jāmiʿ*, *op. cit.* (note 14), vol. 4, 169–70.

31 Ibn Maymūn, *Sharḥ asmāʾ al-ʿuqqār (Commentary on the Names of Drugs)*, in Max Meyerhof (ed.), (Cairo: Institut Français d’Archéologie Orientale, 1940), no. 234.

32 Ibn al-Jazzār, *al-Iʿtimād fī l-adwiya al-mufrada (The Reliable Book on Simple Drugs*), in Idwār al-Qashsh (ed.), (Beirut 1998), 150; cf. Käs, *op. cit.* (note 9), vol. 2, 1068. See also his medical handbook, where *mūmiyāʾ* is mentioned without a specification of its provenience as an ingredient in a recipe: Ibn al-Jazzār, *Zād al-musāfir wa-qūt al-ḥāḍir (Provisions for the Traveller and Nourishment for the Sedentary)*, 2.11.3, in Gerrit Bos , Fabian Käs , and Michael McVaugh (eds), Books I and II: Diseases of the Head and the Face (Leiden: Brill), 306 .

33 Al-Tamīmī, *op. cit.* (note 24), Parisinus arab. 2870, f. 23v.

34 See Karl H. Dannenfeldt , ‘Egyptian Mumia: The Sixteenth Century Experience and Debate’, The Sixteenth Century Journal 16.2 (1985), 163–8011619689 , esp. 170. Cf. Raphael Patai, ‘Indulco and Mumia’, *The Journal of American Folklore* 77 (1964), 3–11, esp. 7–10.

35 On drugs as diplomatic gifts between Byzantine and Islamicate courts, see Koray Durak , ‘Healing gifts: the role of diplomatic gift exchange in the movement of *materia medica* between the Byzantine and Islamicate worlds’, in P. Bouras-Vallianatos and D. Stathakopoulos (eds), Drugs in the Medieval Mediterranean: Transmission and Interaction Across Cultures in Medicine and Beyond (Cambridge: Cambridge University Press, 2023), 388–415 .

36 On Arabic-Greek medical translations, see Alain Touwaide , ‘Agents and agencies? The many facets of translation in Byzantine medicine’, in F. Wallis and R. Wisnovsky (eds), Medieval Textual Cultures (Berlin: De Gruyter, 2016), 13–38 . See also the recent rich critical discussion of Byzantine translations from Arabic into Greek accompanied by evidence from medical texts throughout as well by Maria Mavroudi , ‘Byzantine Translations from Arabic into Greek: Old and New Historiography in Confluence and in Conflict’, Journal of Late Antique, Islamic and Byzantine Studies 2 (2023), 215–88.

37 On diplomatic relations between the Fatimid Caliphate and Byzantium in the tenth century, see Yaacov Lev , ‘The Fatimids and Byzantium, 10^th^-12^th^ centuries’, Graeco-Arabica 6 (1995), 190–208 , esp. 192–203.

38

Paul Magdalino , ‘Pharmaceutical diplomacy: a new document on Fatimid-Byzantine gift exchange’, in T. Antonopoulou et al. (eds), Myriobiblos: Essays on Byzantine Literature and Culture (Berlin: De Gruyter, 2015), 245–51, esp. 245.8–246.11.

39 One Byzantine *litra* was approximately 320 g. Erich Schilbach , Byzantinische Metrologie (Munich: Beck, 1970), 277–8.

40 Magdalino, *op. cit.* (note 38), 246.13–29.

41 A term originally applied to silver coins of 2.27 g., the weight of which was increased later in the Macedonian period to ca. 3.03 g. See Philip Grierson , ‘Miliaresion’, in A. P. Kazhdan (ed.), The Oxford Dictionary of Byzantium, vol. 2 (Oxford: Oxford University Press, 1991), 1373 . Cf. Schilbach, *op. cit.* (note 39), 161.

42 Magdalino, *op. cit.* (note 38), 246.30–2.

43
*Ibid.*, 246.32–5.

44
*Ibid.*, 246.36–40.

45
*Ibid.*, 246.42–4.

46 This is most probably a translation from an Arabic treatise by pseudo-Yūḥannā ibn Māsawayh (d. 857). The earliest surviving manuscript of this text is Vaticanus gr. 300, ff. 273v–284v, where it is attributed to St. John of Damascus. See Bouras-Vallianatos, *op. cit.* (note 3), 987, note 125. This was due to these two historical figures having the same name in Arabic, i.e. Yūḥannā=John. The same confusion is also attested in the Latin tradition, see Paula De Vos, ‘The “Prince of Medicine”: Yūḥannā ibn Māsawayh and the Foundations of the Western Pharmaceutical Tradition’, *Isis* 104.4 (2013), 667–712, esp. 683; on the Latin version of this treatise, see I. Ventura, ‘Sulla trasmissione vernacolare dello «Schriftencorpus» attribuito allo Ps.-Mesue: per una ricognizione delle traduzioni tra XIII e XVI secolo’, *Carte Romanze* 9.2 (2021), 183–265, esp. 185–99.

47 The text was most probably translated into Greek in Southern Italy or Sicily. The *terminus ante quem* for the Greek translation is by 1130/1140, which is the date of the earliest surviving witness, Vaticanus gr. 300. On this translation and its role in disseminating Arabic pharmacological lore in Greek, see Bouras-Vallianatos, *op. cit.* (note 3), 982–7.

48 On the date of this codex, see Paul Canart, ‘Le livre grec en Italie méridionale sous les règnes normand et souabe: aspects matériels et sociaux’, *Scrittura e Civiltà* 2 (1978), 103–62, esp. 146; and Thibault Miguet, ‘Premiers jalons pour une étude complète de l’histoire du texte grec du *Viatique du Voyageur* (Ἐφόδια τοῦ ἀποδημοῦντος) d’Ibn al-Ǧazzār’, *Revue d’Histoire des Textes* 12 (2017), 59–105, esp. 85, 98.

49 Anonymous, *Orneosophion*, in Rudolf Hercher (ed.), *Claudii Aeliani Varia Historia, Epistulae, Fragmenta* (Leipzig: Teubner, 1866), 580.7–9: Περὶ συνθλάσματος ἀπὸ ἐμπλαστῆς ἤτε τζακίσματος ἤ στηθίσματος. Μώμιον μετὰ κρέατος διδόμενον ὠφελεῖ. It is worth noting that the earlier edition of the text by Joseph von Hammer-Purgstall, *Falknerklee, bestehend in drey ungedruckten Werken über die Falknerey* (Pest: Hartleben, 1840), 81.28, gives the variant ‘μωμιά’. On this text, see briefly Herbert Hunger, *Die hochsprachliche profane Literatur der Byzantiner*, vol. 2 (Munich: Beck, 1978), 269. Cf. Stavros Lazaris, ‘La production nouvelle en médecine vétérinaire sous les Paléologues et l’œuvre cynégétique de Dèmètrios Pépagôménos’, in M. Cacouros and M.-H. Congourdeau (eds), *Philosophie et Sciences à Byzance de 1204 à 1453* (Leuven: Peeters, 2006), 225–67, esp. 248–9.

50 Benjamin the Jew, *Recipe Book*, in Petros Bouras-Vallianatos (ed.), *Medieval Greek Recipe Books: Four New Medical Witnesses in Context* (Abingdon: Routledge, forthcoming): Ἔμπλαστρος πρὸς σπάσματα ὄρχεων. Λίβανον· ἀλόην, ἀνὰ στγ. α´· τὰς τρίχας τὰς λεπτὰς τοῦ λαγωοῦ· μώμιον· ἄσφαλτον· αἰγύπτια ῥόδα, ἀνὰ < α´· τὸ λεπτὸν τοῦ γύψου στγ. s´´· στυπτηρίαν στγ. s´´· μαστίχην οὐγγ. β´. The work survives in three manuscripts, with the earliest dated to the fourteenth century. This is also the *terminus ante quem* for Benjamin’s floruit. Ambrosianus Q94 sup., ff. 349r–352v (fifteenth/sixteenth century), 361r–363r; Marcianus Venetus V.8 (coll. 1334) (fourteenth century), ff. 158v–164v; Vaticanus gr. 282 (early fifteenth century), ff. 437v–441v. Erich Trapp, Walther Rainer, and Hans-Veit Beyer (eds), *Prosopographisches Lexikon der Palaiologenzeit*, 11 vols (Vienna: Verlag der Österreichischen Akademie der Wissenschaften, 1976–91), no. 91491. See also Petros Bouras-Vallianatos , Innovation in Byzantine Medicine: The Writings of John Zacharias Aktouarios (c.1275–c. 1330) (Oxford: Oxford University Press, 2020), 145 , note 23.

51 Andreiomenos, *Recipe Book*, Bouras-Vallianatos (ed.), *op. cit.* (note 50): Μούμια πινομένη τρίμμα ἰᾶται δυσεντερικούς. The work survives in an autograph manuscript, Athous Iberiticus 151, ff. 228r–235r, dated to the second half of the fifteenth century.

52

Charles Du Cange , Glossarium ad scriptores mediae et infimae Graecitatis, vol. 2 (Lyon: Anisson; Posuel; Rigaud, 1688), ‘Appendix ad Glossarium’, s.v. ΜΏΜΙΟΝ, 138. Du Cange’s entry was adopted in Henri Stephanus (Estienne), *Thesaurus Graecae linguae* (Geneva, 1572), s.v. Μώμιον, vol. 5, 1333. Emmanouil Kriaras, *Λεξικό της Μεσαιωνικής Ελληνικής Δημώδους Γραμματείας* (Thessaloniki: Kentro Ellinikis Glossas, 1990), s.v. μώμιον, vol. IV, 185–6, refers to it as a kind of pharmaceutical substance of unknown etymology: Άγνωστης ετυμολογίας. Είδος φαρμακευτικής ουσίας. Erich Trapp et al. (eds), *Lexikon zur byzantinischen Gräzität*, 8 vols. (Vienna: Verlag der Österreichischen Akademie der Wissenschaften, 1994–2017), s.v. μώμιον, is similarly mentioned as ‘ein Heilmittel’ (‘a remedy’); cf. *idem*, s.v. μωμία, which is rightly translated as ‘Leichnam’ (‘corpse’) with reference to *Dynameron.*

53 This may also be a place in the area of the Pontos. Anthony Bryer and David Winfield , The Byzantine monuments and topography of the Pontos, vol. 1 (Washington DC: Dumbarton Oaks Research Library and Collection, 1985), 171 , suggest that the castle called Mumya Kale, ca. 160 km southwest of Trebizond, most probably derives its name from the mineral. The area is rich in minerals like copper, but there is no report of *mūmiyāʾ.*

54 In his *Book Most Comprehensive in Knowledge on Precious Stones*, al-Bīrūnī mentions two ways of testing. It may be applied to the liver of an animal. If it is not genuine, the liver will be dissolved. The second method is to break (Said erroneously translated to ‘cut off’) the leg of a chicken. He did not specify what happens next, but because he praised the effectiveness of *mūmiyāʾ* for broken bones, it is clear that the chicken was expected to recover (Bīrūnī, *Jamāhir*, Krenkow (ed.), *op. cit.* (note 17), 206.9; Said (tr.), *op. cit.* (note 17), 178). That both mention chickens is perhaps not coincidental; it is possible that al-Bīrūnī and the alleged Fatimid author depend on a common source. Since this alleged source did not leave clear traces in the classical medical texts examined (see above), we may assume that it also belonged to the genre of geography. The German traveller Engelbert Kaempfer also writes that he himself had experimented in the early eighteenth century with the broken legs of fowls. Since he explicitly states that he had been unable to obtain ‘primary mummy’, it is clear that he did not personally witness the ceremony described above. Rather he may depend on a written source—either al-Bīrūnī or an unnamed geographer. Since al-Bīrūnī mentions a similar way of testing the authenticity of *mūmiyāʾ*, Kaempfer may also have found such a description in this source. Engelbert Kaempfer , Amoenitatum Exoticarum Politico-Physico-Medicarum Fasciculi V, vol. 3 (Lemgo: Typis & impensis Henrici Wilhelmi Meyeri, Aulae Lippiacae, 1712), 516–24. The text is available in German translation by Karl Meier, ‘Über die echte Mumie,’ *Sudhoffs Archiv für Geschichte der Medizin und der Naturwissenschaften* 30.1/2 (1937), 62–9. It has also been translated into English with a brief introduction by Robert W. Carrubba , ‘The First Detailed Report on Persian Mummy’, Physis 32 (1981), 459–7111634381. The Swedish traveller and naturalist Fredrik Hasselquist (1722–1752) also saw and described the mineral mummy during his trips to Egypt and the Middle East in the late 1740s/early 1750s; he also refers to a process of testing similar to that described by Kaempfer, although he states that he had not personally witnessed the procedure being carried out. Hasselquist’s notes were published originally in Swedish by Carl Linnaeus. Here, it was accessed through its English translation, Fredrik Hasselquist, *Voyages and Travels in the Levant in the Years 1749, 50, 51, 52* (London: Davis & Reymers, 1766), 302–4.

55 Arabic sources recommended the internal use of small amounts of *mūmiyāʾ* mixed with diverse beverages for several diseases (e.g. Ibn al-Bayṭār, *Jāmiʿ*, *op. cit.* (note 14), vol. 4, 169f.). One of the earliest Arab physicians, called Abū Jurayj ‘the monk’, stated that the drug was beneficial for fractures both when applied externally and internally (*apud* Ibn al-Bayṭār, *Jāmiʿ*, *op. cit.* (note 14), vol. 4, 170, 10; *apud* al-Rāzī, *al-Ḥāwī*, *op. cit.* (note 14), vol. 21, 498.12; see also Ibn Sīnā, *Qānūn*, *op. cit.* (note 26), vol. 1, 367.30).

56 It is worth noting that Abū Jurayj mentioned that the weight of one carat administered orally along with milk is beneficial for ulcers of the urethra and the bladder (*apud* Ibn al-Bayṭār, *Jāmiʿ*, *op. cit.* (note 14), vol. 4, 170.12). The physicians from Khuzestan recommended *mūmiyāʾ* for incontinence (Ibn al-Bayṭār, *Jāmiʿ*, *op. cit.*, vol. 4, 170.15). Both indications were mentioned by Ibn Sīnā (*Qānūn*, *op. cit.* (note 26), vol. 1, 368.8), who also advised dissolving *mūmiyāʾ* in milk. Also, al-Rāzī in his unedited treatise on *mūmiyāʾ* suggests its use for flatulence and bloating that affects the abdomen (see above, note 25).

57
*Miracles of St. Artemios*, 15, 32, 38, ed. Athanasios Papadopoulos-Kerameus , Varia graeca sacra (St. Petersburg: Kirschbaum, 1909), 15.6, 46.21–2, 62.12–13. This collection was written down in the seventh century.

58 Paul of Aegina, *Epitome*, 5.64, Heiberg (ed.), *op. cit.* (note 8), vol. 2, 107.10–21. A somewhat similar disease was dealt with by al-Rāzī in his monograph *On the Treatment of Small Children*, 23, in Gerrit Bos and Michael McVaugh (eds), *Al-Rāzī, On the Treatment of Small Children* (De curis puerorum)*. The Latin and Hebrew translations* (Leiden: Brill, 2015), 63: ‘Then concerning the stone that is produced in a child’s bladder. Its symptoms are: Strangury, severe pain, thin urine, and itching in the male member, which may be constantly erect’. *Mūmiyāʾ* was, unfortunately, not mentioned among the remedies beneficial for this disease. For a similar description, see Ibn al-Jazzār, *Siyāsat al-ṣibyān wa-tadbīruhum* (*The Care of Children and their Therapy*), in Muḥammad al-Ḥabīb al-Hīlah (ed.), (Tunis: al-Dār al-Tūnisiyya li-l-nashr, 1968), 130–2.

59 See Francis Adams , Paulus Aegineta, vol. 2 (London: Syndenham Society of London, 1844), 371–2, who interprets Paul’s description as an aneurismal varix or erectile tissue.

60 Leo the Physician, *Synopsis of Medicine*, 6.13, in Franz Zacharias Ermerins (ed.), Anecdota medica Graeca e codicibus MSS. expromsit (Leiden: S. et J. Luchtmans, 1840), 197.8–12.

61 Aetios of Amida, *Tetrabiblos*, 15.14, in Skevos Zervos (ed.), ‘Ἀετίου Ἀμιδηνοῦ λόγος δέκατος πέμπτος’, *Ἀθηνᾶ* 21 (1909), 7–138, esp. 59.15–60.11 and 65.4–13.

62 This can be said, for example, of all three Arabo-Greek medical translations mentioned in this article. Ibn al-Jazzār’s *Zād al-Musāfir wa-qūt al-ḥāḍir* provides information about composite drugs, including pharmacological ingredients (e.g. myrobalans, sandalwood, galangal, musk, ambergris, cubeb pepper) from Asia and the Far East, not used before in any Greek or Byzantine work. Al-Rāzī’s (d. c. 925) *Kitāb fī l-Judarī wa-l-ḥaṣba* discusses the treatment of smallpox and measles, on which there were no Byzantine treatises. The work on purgative drugs by pseudo-Yūḥannā ibn Māsawayh introduces a considerable number of simple drugs with purgative effects, such as various kinds of myrobalan, senna, etc., to Byzantine medical literature.

63 See, for example, the case of the late Byzantine practising physician John Zacharias Aktouarios discussed by Bouras-Vallianatos, *op. cit.* (note 50), 149–51.

## Appendix

### Edition and English translation of the Byzantine opuscule on *mūmiyāʾ*

Vaticanus gr. 282 (**V**) is a paper manuscript dated to the early fifteenth century,^64^ has III + 463 folia measuring 294 mm. by 209 mm., with ca. 31 lines to the page. It contains various Galenic works, e.g. *On Mixtures*, *On Anomalous Dyskrasia*, *On Crises*, Books 6–16 of Aetios of Amida’s *Tetrabiblos*, the recipe book of Benjamin the Jew, and various other brief collections of recipes.^65^ Parisinus gr. 2194 (**P**) is dated to the fifteenth century, has III + 465 + II folia, measuring 286 mm. by 210 mm., with ca. 26 lines to the page. It contains Books 5–14 of Aetios of Amida’s *Tetrabiblos* and other collections of recipes, such as the Persian antidotary by Constantine Meliteniotes,^66^ the recipe book of Philip Xeros and Euphemios of Sicily, and two collections of *xenōnika*,^67^ i.e. texts associated with Byzantine *xenōnes* (hospitals).^68^ With the exception of five folia, the manuscript was entirely copied by the physician Demetrios Angelos (c.1430s to early sixteenth century).^69^

The text in question presents only a few variations in the two manuscripts. Vaticanus preserves a longer title, ‘Περὶ τῆς τοῦ μωμίου ἐνεργείας’ (‘On the activity of *mōmion*’) compared to the briefer ‘Περὶ μωμίου’ (‘On *mōmion*’) in Parisinus. Parisinus gives a few more phrases at the beginning outlining the contents of the text than the Vaticanus version, which is rather laconic, does. The following edition is mainly based on Vaticanus, which generally gives better readings, both orthographically and grammatically. Also, the version of Parisinus is sometimes less formal linguistically than that of Vaticanus (e.g. ποτιζούμενον **P**, ποτιζόμενον **V**; ζεστάκρατον **P**, ζεστόν ἄκρατον **V**; καλοκαιρίῳ **P**, θέρει **V**). Accents, including enclitics, and breathings have been tacitly regularised. Iota subscript, which is never used in the manuscripts, has been added. The original punctuation of the manuscripts, which consists of many upper dots and a few commas, has generally been retained. The colon, which marks the end of the title and main body of the text in the manuscript, has been replaced by the modern full stop. The initial letters of toponyms and ethnonyms have been capitalised (e.g. Περσίαν, Ἑλλήνων).

Περὶ τῆς μωμίου ἐνεργείας.

Περὶ τοῦ μωμίου ἐνεργείας οὐχ εὑρίσκομέν τινα τῶν ἀρχαίων ἐξηγητῶν Ἑλλήνων μνημονεύσαντα, ἀλλὰ τοὺς Πέρσας μόνους· καὶ ἐξ αὐτῶν μετέλαβον οἱ τῶν Σύρων ἰατροί· λέγουσι δὲ ὅτι ἀπὸ σπηλαιωδῶν πετρῶν ἱδρώτων γίνεται τῶν εἰς Περσίαν· εὑρίσκεται δὲ πολλάκις καὶ εἴς τινας τόπους Ῥωμαϊκούς, ἀλλ᾽ οὐχ ὡς ἐκεῖνο καλόν· οὕτε τὴν πεῖραν αὐτοῦ ἔχουσιν, ὥσπερ οἱ Πέρσαι· τὸ δὲ καλὸν καὶ ἐνεργέστερον αὐτοῦ ἐκ τῆς πείρας μανθάνομεν· πλὴν τὸ μέσον τοῦ τε σκληροῦ καὶ τοῦ πάνυ λείου ἐστὶ τὸ κάλλιστον καὶ χροίαν ἔχον οὐ πάνυ μέλαιναν· δοκιμάζεται δὲ εἰς θραῦσμα ὀρνιθοπούλου ποτιζόμενον ἐξ αὐτοῦ ὡσεὶ κοκκίον ξυλοκεράτου, λυθὲν εἰς οἶνον ζεστὸν ἄκρατον στγ. α´· καὶ δεθέντος τοῦ θραύσματος εἰ μὲν εἰς γ´ ἢ δ´ ἡμέρας περιπατήσει, ἔνι καλὸν τέλειον· ἐὰν δὲ ἀνθρώπου θραυσθῇ ἢ χεὶρ ἢ ποὺς ἢ ἄλλο τι, καὶ θέλῃ πιεῖν· εἰ μέν ἐστι παιδίον μικρὸν γαλουχούμενον, ὀφείλει λαβεῖν ὡσεὶ κοκκίον κριθῆς, λυθὲν μετὰ γάλακτος τῆς μητρὸς αὐτοῦ· εἰ δὲ πενταετές, κοκκίον ξυλοκεράτου· καὶ καθ᾽ ἡλικίαν· εἰ δὲ τέλειος ἀνὴρ εἴη, κοκκία ς´ ἐν χειμῶνι μετὰ οἴνου παλαιοῦ ζεστοῦ ἀκράτου· εἰ δ᾽ ἐν θέρει, κοκκία δ´· εἰ δὲ γέρων εἴη, καὶ πλέον· ποτίζουσι δὲ καὶ μικρὰ παιδία, εἴ γε ὀγκωθεῖεν τὰ αἰδοῖα αὐτῶν· καὶ τηνικαῦτα παύει οὐ μόνον δὲ τὰ παιδία, ἀλλὰ καὶ ἄνδρας, εἴ γε γνώσειε πρὸ τοῦ ὅλως τὸ πνεῦμα κατελθεῖν καὶ ὀγκωθεῖεν.

1 Περὶ τῆς μωμίου ἐνεργείας V: Περὶ μωμίου P || 2 Περὶ τοῦ μωμίου ἐνεργείας, οὐχ V: Τί ἐστι μώμιον καὶ πόθεν γίνεται· καὶ ποῖόν ἐστι κρεῖττον· τὸ λεῖον ἢ τὸ σκληρότερον· καὶ ποῦ ἐνεργεῖ· καὶ πῶς ποτίζεται· περὶ τούτου οὐχ P || 3 τοὺς Πέρσας μόνους V: μόνους τοὺς Πέρσας P || 4 τινας P: οm. V || 7 μέλαιναν V: μέλαν P | ποτιζόμενον V: ποτιζούμενον P || 8 κοκκίον scripsi: κοκίον P: κοκ V | ζεστὸν ἄκρατον V: ζεστάκρατον P | γ´ V: τρεῖς P || 9 δ´ V: τέσσαρας P | περιπατήσει V: πατήσει P || 9-10 ἐὰν δὲ…πιεῖν V: καὶ ἐξ αὐτοῦ ἐὰν ἄνθρωπος θραυσθεὶς θέλει ποιεῖν P || 10 γαλουχούμενον V: χαλουχούμενον P | ὀφείλει scripsi: ὀφελ V: ὠφείλει P || 11 πενταετές P: προταετές V || 11-12 εἰ δὲ τέλειος ἀνὴρ εἴη V: τῷ δὲ τελείῳ ἀνδρί P || 12 ζεστοῦ ἀκράτου V: ζεστακράτου P | θέρει V: καλοκαιρίῳ P | κοκκία V: οm. P || 12-13 εἰ δὲ γέρον εἴη, καὶ πλέον V: ἐν δὲ γέροντι, πλειότερον P || 13 ὀγκωθεῖεν V: ὀγκοθεῖεν P || 14 γνώσειε V: γνώσειεν P | ὀγκωθεῖεν V: ὀγκοθεῖεν P ||

On the activity of *mōmion*

We do not find any ancient Greek commentator referring to the activity of *mōmion*, only the Persians.^70^ And the Syrian^71^ doctors received it from them [i.e. the Persians]. They say that [*mōmion*] is produced from sweating rocks in caves found in Persia. It is also often found in some Roman places,^72^ but it is neither as good as the former, nor do they^73^ have the same experience of it as the Persians. We have learnt from experience that [the Persian] one is better and more efficacious than that one [i.e. the one found in the Roman places]. One balanced between hard and very smooth is the best; and one whose colour is not too black. It can be tested on a fracture in a young chicken, which should drink [a quantity of] just one carob seed^74^ of it, dissolved in one stagion^75^ of unmixed hot wine. And if, once the fracture is bandaged, it walks in three or four days, [the quality of *mōmion*] is perfectly good. If a human hand, foot, or some other [bodily] part is broken, [the patient] needs to drink it as follows: if it is a little breastfed child, it needs to take [a quantity of] just a barley seed, dissolved in the milk of its mother. If [the child] is five years old, [the dosage is] one carob seed; and then the dosage should be adjusted according to the age. If [the patient] is an adult male, [the dosage is] six [carob] seeds taken with hot, unmixed, old wine in wintertime. If it is summertime, [the dosage is] four [carob] seeds. If [the patient] is an old man, give more. They also give it to little children to drink, if their genitals are swollen; and it immediately cures not only children, but also adult males, if [the patient] notices the gas before it goes down entirely and becomes swollen.

